# Guided Internet-Based Cognitive Behavioral Therapy for Depression: Implementation Cost-Effectiveness Study

**DOI:** 10.2196/27410

**Published:** 2021-05-11

**Authors:** Jordi Piera-Jiménez, Anne Etzelmueller, Spyros Kolovos, Frans Folkvord, Francisco Lupiáñez-Villanueva

**Affiliations:** 1 Open Evidence Research Group Universitat Oberta de Catalunya Barcelona Spain; 2 Servei Català de la Salut Barcelona Spain; 3 Digitalization for the Sustainability of the Healthcare System Sistema de Salut de Catalunya Barcelona Spain; 4 GET.ON Institute GmbH/HelloBetter Hamburg Germany; 5 Nuffield Department of Orthopaedics Rheumatology and Musculoskeletal Sciences University of Oxford Oxford United Kingdom; 6 Department of Communication and Cognition Tilburg School of Humanities and Digital Sciences Tilburg University Tilburg Netherlands; 7 Department of Information and Communication Sciences Universitat Oberta de Catalunya Barcelona Spain

**Keywords:** digital health, telemedicine, eHealth, e–mental health, internet-based cognitive behavioral therapy, depression, iCBT, implementation, cost-effectiveness, cognitive behavioral therapy, CBT, cost

## Abstract

**Background:**

Major depressive disorder is a chronic condition; its prevalence is expected to grow with the aging trend of high-income countries. Internet-based cognitive-behavioral therapy has proven efficacy in treating major depressive disorder.

**Objective:**

The objective of this study was to assess the cost-effectiveness of implementing a community internet-based cognitive behavioral therapy intervention (Super@, the Spanish program for the MasterMind project) for treating major depressive disorder.

**Methods:**

The cost-effectiveness of the Super@ program was assessed with the Monitoring and Assessment Framework for the European Innovation Partnership on Active and Healthy Ageing tool, using a 3-state Markov model. Data from the cost and effectiveness of the intervention were prospectively collected from the implementation of the program by a health care provider in Badalona, Spain; the corresponding data for usual care were gathered from the literature. The health states, transition probabilities, and utilities were computed using Patient Health Questionnaire–9 scores.

**Results:**

The analysis was performed using data from 229 participants using the Super@ program. Results showed that the intervention was more costly than usual care; the discounted (3%) and nondiscounted incremental cost-effectiveness ratios were €29,367 and €26,484 per quality-adjusted life-year, respectively (approximately US $35,299 and $31,833, respectively). The intervention was cost-effective based on the €30,000 willingness-to-pay threshold typically applied in Spain (equivalent to approximately $36,060). According to the deterministic sensitivity analyses, the potential reduction of costs associated with intervention scale-up would reduce the incremental cost-effectiveness ratio of the intervention, although it remained more costly than usual care. A discount in the incremental effects up to 5% exceeded the willingness-to-pay threshold of €30,000.

**Conclusions:**

The Super@ program, an internet-based cognitive behavioral therapy intervention for treating major depressive disorder, cost more than treatment as usual. Nevertheless, its implementation in Spain would be cost-effective from health care and societal perspectives, given the willingness-to-pay threshold of €30,000 compared with treatment as usual.

## Introduction

Population aging is a global trend and is expected to be one of the most significant societal challenges worldwide in upcoming years [1]. The profound impact that this aging trend is likely to cause on our societies and economies has prompted significant efforts in turning the challenges of this scenario into opportunities for rethinking the way we design and organize our society, including the delivery of health and social care services [2-5].

Depression is a significant contributor to morbidity during entire lifespans and has been among the 3 leading nonfatal causes of disability globally for nearly three decades [6]. Although often underdiagnosed, depression is the most prevalent mental health condition among adult population and across cultural settings resulting in an aggregate point prevalence of 12.9%, 1-year prevalence of 7.2%, and lifetime prevalence of 10.8% (years 1994-2014) [7-9].

The burden of depression is specifically high among the elderly, irrespective of the presence of cognitive impairment, particularly in long-term care settings [8,10,11]. Various factors may increase the risk of depression among older people, including physiological factors (eg, cardiovascular disease, diabetes, or immunological changes) and psychosocial factors (eg, low economic status, social isolation, or relocation) [12-14]. Once established, depression in older people increases the risk of suicide and may trigger dementia [10].

While the efficacy of psychotherapy in the treatment of depression has been proven [15], the availability of evidence-based interventions constitutes a persistent challenge given the lack and unequal distribution of qualified practitioners, delayed provision of treatment, and inadequacy of treatment [16,17]. Given the limitations and health care costs associated with treating depression (eg, US $7638, according to a study conducted in Singapore [18]), there is growing interest in alternative therapies to routine care. Among them, a plethora of internet-based cognitive behavioral therapies for depression treatment have been introduced, many showing efficacy in treating major depressive disorder [19-21]. Although costs associated with the implementation of these therapies have been assessed, most studies are based on descriptive approaches, and formal cost-effectiveness analysis of internet-based cognitive behavioral therapy interventions are scarce [22].

While randomized controlled trials and accompanying cost-effectiveness analysis can be considered the gold standard in exploring the cost-effectiveness of mental health interventions, the idealized and controlled nature of these trials limits the generalizability of findings to routine care populations [23-25]. Establishing the cost-effectiveness of an intervention and its implementation under routine care conditions is an important part of the evaluation before wide-scale adoption. So far, establishing the cost-effectiveness of implementation projects in routine care provides a methodological challenge.

MasterMind was a European cofunded project aimed at scaling-up the implementation of evidence-based internet interventions (eg, internet-based cognitive behavioral therapy) for the treatment of adults experiencing depressive symptoms across Europe [26]. In this study, we assessed the cost-effectiveness of the Super@ intervention as part of its implementation within the MasterMind project in a pilot site in Spain. The current analysis was performed using the Monitoring and Assessment Framework for the European Innovation Partnership on Active and Healthy Ageing (MAFEIP) tool, developed for monitoring the financial sustainability of initiatives for promoting a healthy lifespan of European citizens [27,28]. Provided as a free-to-access tool for economic evaluations, MAFEIP has gained relevance over the years, and its usage is expanding, particularly within the European project landscape.

## Methods

### Overview of Study Design

As part of the MasterMind project for implementing an internet-based cognitive behavioral therapy for treating depression, we designed a pragmatic within-group trial to assess the cost-effectiveness of the intervention [29,30]. The evaluation framework applies the Model for Assessment of Telemedicine applications [31], which helped to define the data collection tools and instruments according to 3 levels of stakeholders involved within the implementation process: (1) patients, (2) professionals, and (3) organizations.

This analysis corresponds to the experience of the MasterMind project in the BSA (*Badalona Serveis Assistencials*) consortium, implemented under a program named *Super@ tu depresión* (“Get over your depression”). The BSA consortium provides primary and specialized care to a catchment population of 433,175 inhabitants in the most densely populated suburban area of Barcelona and has a long tradition in integrated care and the adoption of digital health solutions [32-38]. The implementation and data collection process for the Badalona pilot site was carried out between March 2015 and June 2017. The outcomes and costs of the intervention were compared with those of usual care in previously published data from the same area [39].

The local study protocol was approved by the Ethics Committee of the Hospital *Germans Trias i Pujol* (reference PI-15-069), and all participants provided informed consent before entering the study.

### Participants

Study candidates included health care recipients and were screened for eligibility after general practitioner referral in the primary care setting. All consecutive patients who visited their general practitioners during the study period and met the selection criteria were offered the opportunity to participate in the Super@ program. Patients included in the study were adults (ie, 18 years or older) diagnosed with mild, moderate, or severe major depressive disorder based on the Patient Health Questionnaire 9 (PHQ-9; with score cutoffs of 10, 15, and 20 for mild, moderate, and severe major depressive disorder, respectively), living in Badalona and who, according to their general practitioner, had a certain level of technological literacy and internet connection. The main exclusion criteria were having comorbidities that may interfere with the treatment, having a nonpsychiatric disease that could explain depressive symptoms, receiving structured face-to-face psychological therapy at the time of inclusion, and reporting a high suicidal risk or ideation (item 9 of the PHQ-9). After checking all selection criteria and obtaining written informed consent, the general practitioner referred participants to the internet-based cognitive behavioral therapy service, provided a comprehensive explanation about the intervention, and enrolled participants in the platform, which automatically provided a username and a password to the participant.

### Intervention

The Super@ program (Multimedia Appendix 1) consisted of 9 modules (8 regular and 1 extra) composed of videos, text content, and questionnaires to monitor the progression of symptoms and adherence to the intervention. Therapists provided guidance and project management within the BSA team to ensure patient follow-up and activation of the appropriate resources upon an increase of depressive symptoms. A project management team facilitated the project and its implementation process. Table S1 (Multimedia Appendix 1) summarizes the main activities performed in the project and the different professional profiles and teams involved in each. Intervention completion (ie, minimal adequate dose) was defined as engaging in a minimum of 3 modules of internet-based cognitive behavioral therapy.

### Cost-Effectiveness Assessment

#### Model Structure, Transition Probabilities, and Utility Estimates

**Figure 1:**
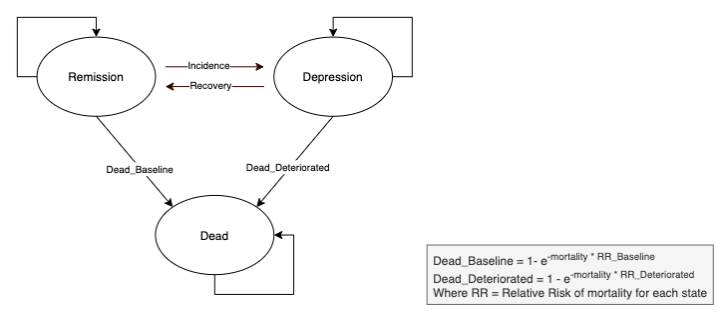
Diagram of the 3-state Markov model of health states and transition probabilities designed for the Super@ intervention.

The cost-effectiveness of the Super@ program was assessed using the MAFEIP tool, which computes costs and utilities using a Markov model of health states and corresponding transition probabilities [40]. Based on previous economic evaluations of treatments for major depressive disorder, we defined a 3-state Markov model, with remission (PHQ-9 score <10), depression (PHQ-9 score ≥10), and death [41] (Figure 1). Transitions between the 3 states of the Markov model included recovery (ie, the probability of going from depression to remission) and relapse (ie, the probability of going from remission to depression); death was used as an absorbing state. The transition probabilities for the intervention group were calculated based on the changes between the health states at baseline and after the intervention. Given the lack of a control group, the corresponding probabilities for treatment as usual were obtained from a recent meta-analysis [41] assessing the usual care effects on major depressive disorder, which included 38 studies with pooled a remission rate (adjusted for publication bias) of 33% (95% CI 26%-40%). As suggested elsewhere [42], the risk of all-cause mortality was derived from life tables―in this case, the Human Mortality Database [43], which is stratified by gender and provides mortality rates at concrete years of age―and adjusted for depression [44].

In accordance with standard guidelines for estimating quality-adjusted life-years in economic evaluations, the MAFEIP tool recommends computing utilities based on measures of health-related quality of life, preferably the EuroQoL 5-dimension (EQ-5D) questionnaire [45]. However, no estimates of health-related quality of life were collected during the assessment of the Super@ program. Alternatively, based on the relationship between utility scores derived from (including the EQ-5D score) and depression health states reported by Kolovos et al [46], we estimated the remission utility from the results of the PHQ-9 measure: the values proposed for 4 clinical categories of major depressive disorder severity were adapted to the 3-state model by estimating the weighted average of utilities of patients in the remission state (ie, PHQ-9 score <10) and those in the depression state (PHQ-9 score ≥10) (Multimedia Appendix 1).

#### Cost Estimate

The MAFEIP tool considers 3 types of costs: one-off costs, which represent the total cost incurred only once at the implementation point (ie, implementation support, training provision of professional staff, and cost of devices), health care costs (ie, health care resources consumption such as costs associated with the time spent by health care professionals on service provision, hospitalizations, pharmacy, etc), and societal costs (ie, related to the time spent by either patients or informal caregivers such as the time spent using the technology or traveling to the hospital).

One-off costs were the main costs of implementing the Super@ services and included the support given to therapists to implement the intervention in their daily routine, the training of professional users, and the costs of development and adaptation of Super@ to the existing information and communication technology platform. Based on the annual gross salary of technical staff in Spain and the number of hours devoted to the project (ie, part-time 50%), we estimated €158 per patient (approximately US $190; an exchange rate of approximately €1 to US $1.20 is applicable at the time of publication). The costs of development and adaptation of Super@ to the existing information and communication technology platform were €237 per patient (US $285). Recurring costs, which included direct costs of each internet-based cognitive behavioral therapy session, amounted to €2439 (US $2927) per patient. For the control group, the typical situation is setting the one-off and recurrent costs at 0, because in most cases, the intervention would mean an additional investment.

Health care and societal costs were not collected in the MasterMind project. These costs were gathered from a previous study [39] that described the costs associated with major depressive disorder in the same area. Based on this study [39], we established the health care costs for patients in remission and depression as €451 and €826, respectively (US $542 and US $993). Correspondingly, the costs due to loss of labor productivity were €991 and €1842 for patients in remission and depression, respectively (US $1191 and US $2214). Health care and societal costs were assumed to be the same for the intervention and control groups.

In accordance with recommendations from local health technology assessment authorities in Spain, we applied a discount factor of 3% for both health care outcomes and costs [47]. The willingness-to-pay threshold was established at €30,000 per quality-adjusted life-year, the threshold most frequently used in Spain (equivalent to approximately $36,060).

The cycle length of the Markov model was set at 1 year (ie, the maximum allowed in the MAFEIP tool). Given the chronic nature of major depressive disorder [48], we established the number of cycles necessary to cover the time lapse between the average age of study participants (ie, 46 years) and a theoretical lifespan time of 100 years.

#### Analysis

The cost-effectiveness analysis was performed on the intention-to-treat sample, which included all participants who started at least 1 module of the treatment. The clinical and demographic characteristics of study participants were described with R software (version 3.5.3; The R Project) using the frequency percentage and the mean and standard deviation for categorical and quantitative variables, respectively. Variables of time were described as the median and interquartile range. The cost-effectiveness analysis was conducted using the MAFEIP tool including health states, transition probabilities, utility scores, and costs. All participants started on the state *depression* in the 3-state Markov model.

In addition to the base-case analysis, we conducted deterministic sensitivity analyses for 2 scenarios: reduction in session cost (up to 25%) associated with a lower professional-to-patient ratio expected for a scaling up of the intervention, and 0% to 5% discount in utilities, as recommended by local guidelines for economic evaluations [49]. Sensitivity analyses were nondiscounted.

Transition probabilities were computed using R software, whereas costs and utilities were calculated using a spreadsheet (Excel, version 2013; Microsoft Inc).

## Results

### Study Population and Intervention Conduct

Of the 253 patients recruited for the study, 229 participants (90.5%) started at least one module of the treatment (intention-to-treat sample), of whom 1 participant (0.4%) did not provide data on posttreatment status, and 81 participants (35.4%) did not complete treatment; therefore, 147 participants completed the treatment (Multimedia Appendix 1). All participants had been recruited during a clinical interview after referral by their general practitioner, and all completed the PHQ-9 questionnaire. Table 1 summarizes demographic and clinical characteristics of the intention-to-treat sample at baseline. Participants in the intention-to-treat sample remained under the Super@ program a median of 96 days (IQR 70-321); 147 participants (64.2%) were considered to have completed the study. At the end of the intervention, 98 participants (66.7%) had achieved the remission state. No adverse events related to the intervention or the major depressive disorder were reported.

**Table 1:** Clinical and demographic baseline characteristics of the participants who started the treatment.

Characteristic		Intention-to-treat sample (n=229)
Age (years), mean (SD)		46.40 (12.51)
**Gender, n (%)**		
	Male	73 (31.9)
	Female	156 (68.1)
**Education, n (%)**		
	Primary	42 (18.3)
	Secondary	100 (43.7)
	Higher	78 (34.1)
	Other	8 (3.5)
	Not answered	1 (0.4)
**Employment, n (%)**		
	Yes	169 (73.8)
	No	58 (25.3)
	Unknown	1 (0.4)
	Not answered	1 (0.4)
**Depressive episodes, n (%)**		
	Less than 4 weeks	10 (4.4)
	Between 4 and 8 weeks	40 (17.5)
	Between 8 and 12 weeks	65 (28.4)
	Between 3 and 6 months	51 (22.3)
	Between 6 months and 1 year	36 (15.7)
	Between 1 year and 3 years	23 (10.0)
	Between 3 and 5 years	2 (0.9)
	Between 5 and 10 years	2 (0.9)
**Antidepressant medication, n (%)**		
	Yes, for less than 1 month	7 (3.1)
	Yes, for less than 2 months	44 (19.2)
	Yes, for more than 2 months	74 (32.3)
	No	104 (45.4)
**Satisfaction with life^a^, n (%)**		
	Preintervention	3.50 (1.16)
	Postintervention	4.03 (1.28)
**Satisfaction with mental health^a^, n (%)**		
	Preintervention	3.23 (1.03)
	Postintervention	3.98 (1.32)

^a^Assessed using a single-item question (How satisfied are you with your life as a whole today? or How satisfied are you with your mental health?) and rated on a 6-point scale (1=couldn’t be worse, 2=displeased, 3=mostly dissatisfied, 4=mixed, 5=mostly satisfied, 6=pleased, 7=couldn’t be better).

### Study Parameters and Base Case Analysis

Table 2 summarizes the inputs of the cost-effectiveness analysis, including transition probabilities, costs, and utilities.

The Super@ program cost more than usual care from both health care and societal perspectives (Table 3).

**Table 2:** Inputs of the MAFEIP tool for computing the cost-effectiveness analysis.

Input		Control group	Intervention group (n=229)
**Transition probabilities (%)**			
	Remission	14	0
	Depression	29	48.53
Costs (€^a^ per patient and year)			
One‐off cost per patient		N/A^b^	395.26
Recurring cost per patient per year		N/A	2439
**Health care cost**			
	Remission	451	451
	Depression	826	826
**Societal cost**			
	Remission	991	991
	Depression	1842	1842
**Utilities**			
	Remission	0.62	0.665
	Depression	0.532	0.529
**Relative risk of mortality**			
	Remission	1	1
	Depression	1.68	1.68

^a^An exchange rate of approximately €1 to US $1.20 is applicable at the time of publication.

^b^N/A: not applicable.

**Table 3:** Incremental costs, effects, and cost-effectiveness ratio from health care and societal perspectives.

Perspective		Incremental cost (€^a^)	Incremental effects (QALY^b^)	Incremental cost-effectiveness ratio (€/QALY)
**Health care perspective**				
	Discounted (3% for both costs and effects)	50,924.53	1.734	29,366.92
	Nondiscounted	87,807.06	3.315	26,484.27
**Societal perspective**				
	Discounted (3% for both costs and effects)	48,178.53	1.734	27,783.38
	Nondiscounted	83,181.81	3.315	25,089.21

^a^An exchange rate of approximately €1 to US $1.20 is applicable at the time of publication.

^b^QALY: quality-adjusted life-year.

**Figure 2:**
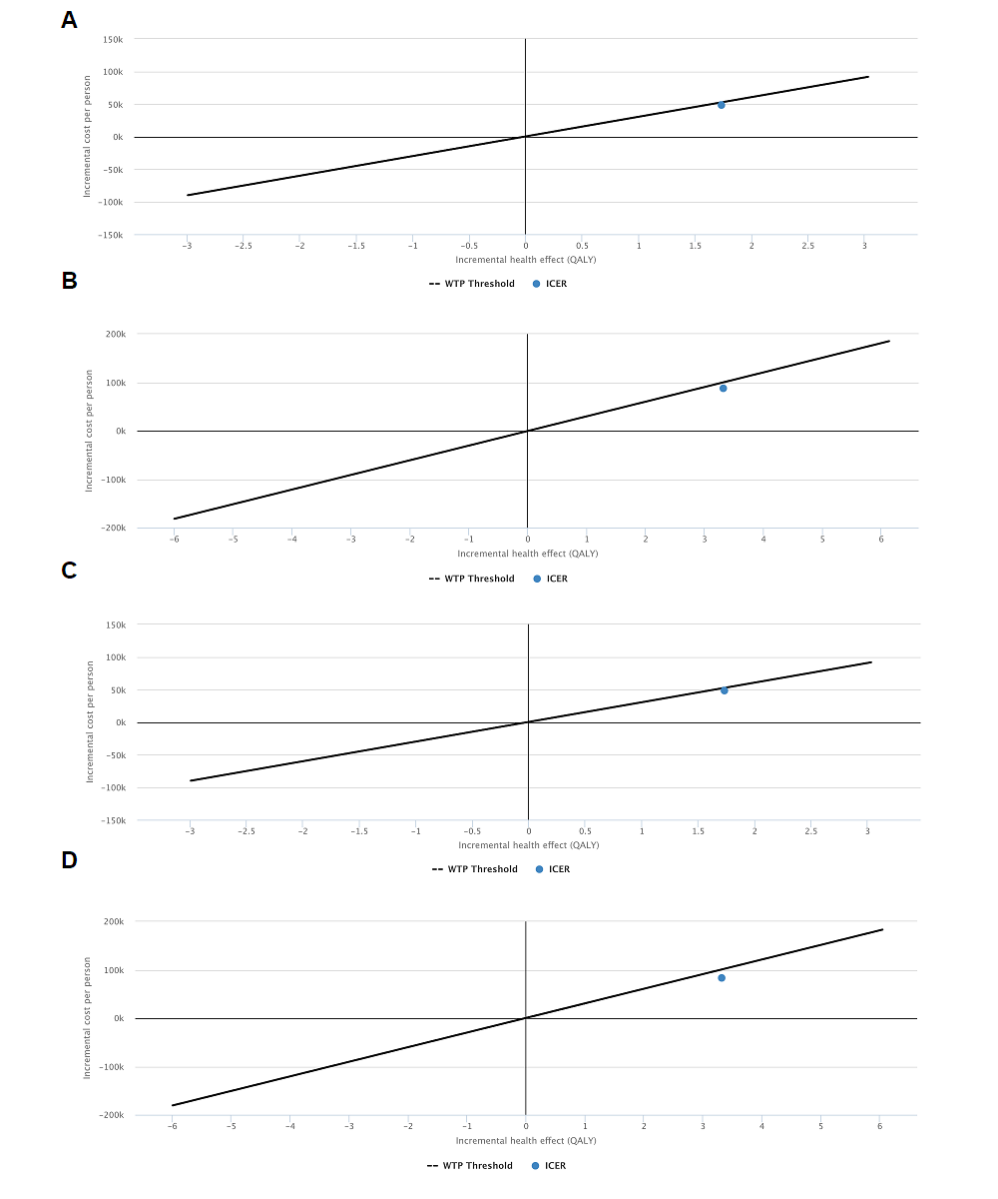
Cost-effectiveness plane of the Super@ intervention Healthcare perspective discounted (3% for both costs and health effects) (A) and non-discounted (B) analyses. Societal perspective discounted (3% for both costs and health effects) (C) and non-discounted (D) analyses. The solid line shows the 30,000 €/QALY willingness-to-pay threshold (equivalent to approximately US $36,060; an exchange rate of approximately €1 to US $1.20 is applicable at the time of publication). QALY: quality-adjusted life-year. WTP: willingness-to-pay.

The nondiscounted incremental cost-effectiveness ratios were below the willingness-to-pay threshold of €30,000 (Figure 2): €26,484 and €25,089 for health care and societal perspectives, respectively (US $31,833 and $30,162). The discounted incremental costs and effects were higher, although the incremental cost-effectiveness ratios remained below the willingness-to-pay threshold of €30,000.

**Figure 3:**
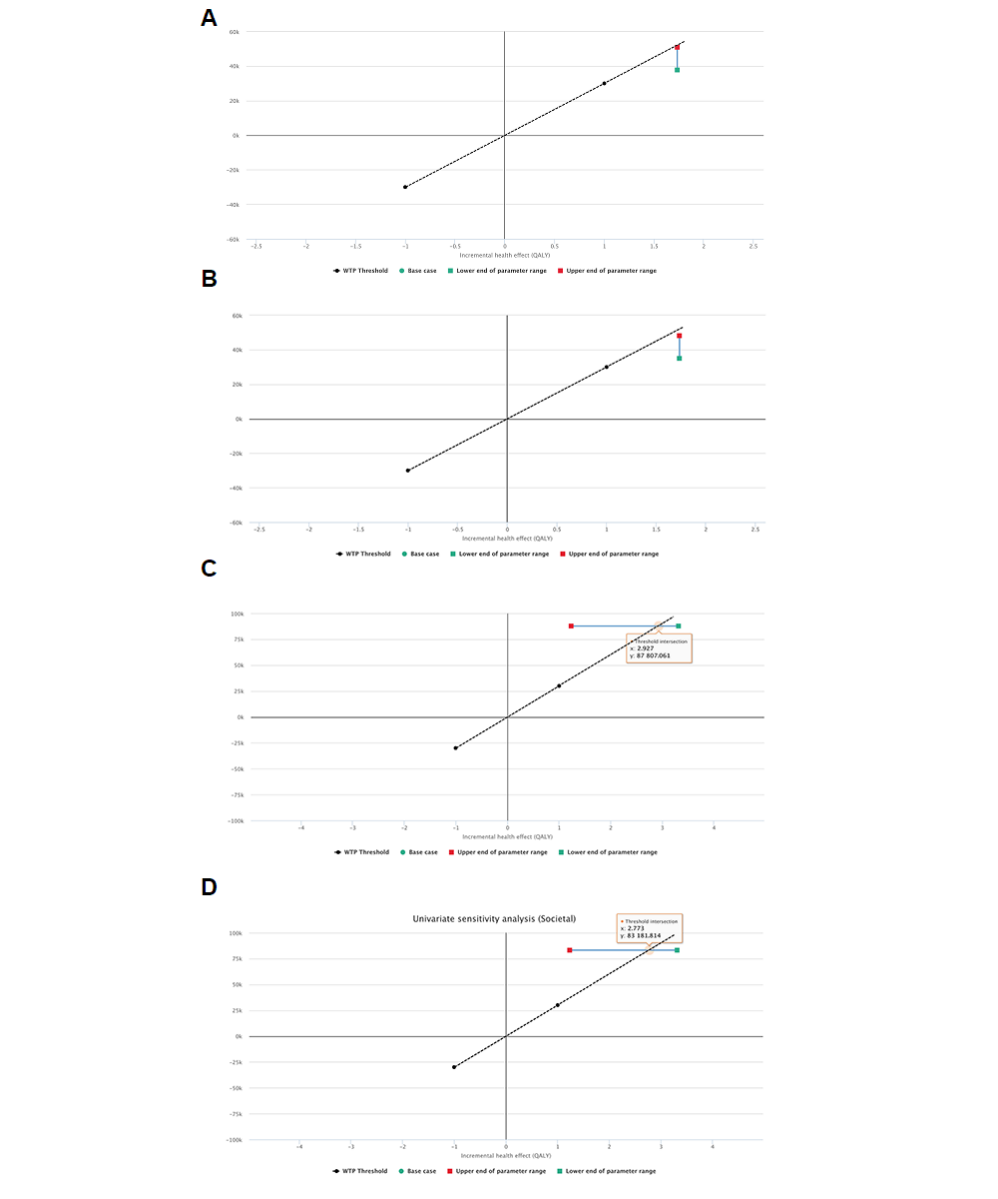
Cost-effectiveness planes of sensitivity analyses. A reduction of up to 25% in cost per session (A and B for healthcare and societal perspectives, respectively), and 0% to 5% discount in effects (C and D for healthcare and societal perspectives, respectively). The dotted black line shows the 30,000 €/QALY willingness-to-pay threshold (equivalent to approximately US $36,060; an exchange rate of approximately €1 to US $1.20 is applicable at the time of publication). The solid green line shows the range of the incremental cost-effectiveness ratio, with the red and green squares indicating the range extremes for the worse (more costly or less effective) and best (less costly or more effective) scenario, respectively. QALY: quality-adjusted life-year. WTP: willingness-to-pay.

In addition, we conducted a deterministic sensitivity analysis assuming that a greater number of participants to the program would results in a reduction of cost per session. A 25% reduction in the cost per session would reduce the incremental cost-effectiveness ratio from €26,484 to €19,623 (US $31,833 to $23,591) in the health care perspective analysis and from €25,089 to €18,228 (US $30,162 to $21,914) in the societal perspective analysis (Figure 3A and 3B). From the health care perspective (Figure 3C), the incremental cost-effectiveness ratio at the 5% discount in utility (worst-case scenario of the sensitivity analysis) was €71,041 (US $85,405). The corresponding intersection and lowest incremental cost-effectiveness ratio values for the societal perspective were 2.773 quality-adjusted life-years and €30,000 (Figure 3D).

## Discussion

In this analysis of the cost-effectiveness of an internet-based cognitive behavioral therapy intervention for mild or moderate major depressive disorder, we found that the intervention was more effective than treatment as usual, with incremental costs of €87,807 and €83,181 (nondiscounted from the health care and societal perspectives, respectively; US $105,561 and $99,999), according to costs reported for routine care of patients with mild-to-moderate major depressive disorder in our area (Badalona, Spain). Despite the higher cost of the internet-based cognitive behavioral therapy intervention, it remained below the willingness-to-pay threshold of €30,000 typically used in Spain for making decisions in health care policies. According to the sensitivity analyses, the internet-based cognitive behavioral therapy would remain more expensive and more effective than treatment as usual in the onset of the cost reduction expected when scaling up the intervention (with the consequent decrease of the professional-to-patient ratio), with an incremental cost-effectiveness ratio below the willingness-to-pay threshold. When considering a 5% reduction in utility (ie, as suggested by local guidelines for economic evaluations), the intervention remained more effective than treatment as usual, although with an incremental cost-effectiveness ratio above the willingness-to-pay threshold.

In the last decade, many studies [50-53] have investigated the costs associated with internet-based cognitive behavioral therapy interventions, including therapies for major depressive disorder; however, most are based on descriptive approaches, which preclude drawing conclusions that can be used for making decisions on their implementation. More recently, Paganini et al [22] reviewed economic evaluations of internet-based cognitive behavioral therapy interventions for major depressive disorder that fulfilled preselected quality criteria, including the presence of comparator groups such as treatment as usual, another intervention, or wait-list controls. The case-mix of these interventions and heterogeneity of analyses precludes direct comparisons regarding the cost-effectiveness of each intervention. Nevertheless, they found that guided interventions (such as the Super@ program) tended to be more cost-effective than self-help ones, despite the higher cost associated with professional honoraria [22]. The incremental cost-effectiveness ratio of our intervention for the base-case health care perspective (€26,484 per quality-adjusted life-year) was in the lower zone of the wide range of values reported for guided interventions (ie, €19,616 [54] to €157,900 [55]; approximately US $19,616 and $189,825, respectively) and below that of unguided interventions (ie, €40,412 [56] to €178,700 [57]; approximately US $48,583 and $214,831, respectively).

Additionally, such studies [51,52] can only report on cost-effectiveness measures in controlled settings. Our study focused on the assessment of cost-effectiveness under real-world conditions free from biases possibly being introduced within efficacy studies such as a stricter application of protocolized procedures, eligibility criteria, and randomization [23-25]. Nevertheless, this approach resulted in some disadvantages, and our results should be interpreted with caution due to several limitations.

The lack of a comparator group has been considered among the limitations of economic evaluations of internet-based cognitive behavioral therapy interventions for major depressive disorder [22]. The pragmatic approach of our study, which took advantage of the implementation of the Super@ program by the local health care provider to assess its cost-effectiveness, precluded collecting treatment-as-usual data in parallel with those collected for the internet-based cognitive behavioral therapy intervention; however, the MAFEIP tool allowed us to rely upon literature for gathering these data. Of note, the source of cost-estimate data of treatment as usual for major depressive disorder corresponded to the same area in which the Super@ program was deployed [39]. Hence, the costs attributed to treatment as usual are expected to be similar to those we would have observed in a control group.

The MAFEIP tool also allowed us to bypass the unavailability of EQ-5D scores of health-related quality of life, a widely accepted measurement for computing utilities in cost-effectiveness analyses [45,58]. Other measures, such as disease severity scores, have been proposed as a proxy for health-related quality of life [59]. Taking advantage of the analysis by Kolovos et al, who established a relationship between health-related quality of life and PHQ-9 score for major depressive disorder severity [46], we computed the utility of the remission state of our 3-state Markov model using the PHQ-9 scores at the cutoff for minor depressive symptoms in the 5-state scale defined by the American Psychiatric Association [60] and the National Institute for Clinical Excellence [61].

Readers should take into consideration some limitations of the study design. First, the pragmatic approach constrained the number of participants to the implementation capacity, rather than the adequate sample size to achieve precision in our estimates. Second, like many other information and communication technology-based solutions, the success of an internet-based cognitive behavioral therapy intervention requires minimal technological literacy, which in our intervention was measured in an unstructured way at each general practitioners discretion. Technological literacy and keenness for the use of digital gadgets are expected to influence not only adherence but also the benefit that the patient may obtain from the intervention; the unstructured assessment of digital literacy may have introduced heterogeneity in the intervention outcomes. Third, the transferability of the results to other settings should be considered carefully. There are many reasons why cost-effectiveness analysis of health technologies may differ across jurisdictions and researchers and implementers should always refer to national guidelines in order to shed some light on the applicability of the results emerging from other contexts [62].

Our results suggest that the Super@ program provided benefits to patients at a cost that would allow its implementation in Spain, where interventions below €30,000 per quality-adjusted life-year are accepted. Costs associated with the intervention are expected to decrease in a scaling-up scenario; however, the sensitivity analysis of utility indicates that small reductions in effects would place the intervention at a nonacceptable incremental cost-effectiveness ratio based on the €30,000 threshold. Future studies should explore the patient profile that can benefit most from the intervention so that general practitioners have more information to target the therapy adequately.

## Appendix

Multimedia Appendix 1 Supplementary file 1.

## Abbreviations

BSA: Badalona Serveis Assistencials
EQ-5D: EuroQoL 5-dimension questionnaire
MAFEIP: Monitoring and Assessment Framework for the European Innovation Partnership (on Active and Healthy Ageing).
PHQ-9: Patient Health Questionnaire–9
