# Supplementary material for: Guided Internet-Based Cognitive Behavioral Therapy for Depression: Implementation Cost-Effectiveness Study
Source: J Med Internet Res. 2021 May 11;23(5):e27410. doi: 10.2196/27410 (PMC8150403; doi:10.2196/27410)

Jordi Piera-Jiménez, Anne Etzelmueller, Spyros Kolovos, Frans Folkvord, Francisco Lupiáñez-Villanueva

Guided Internet-Based Cognitive Behavioral Therapy for Depression: Implementation Cost-Effectiveness Study

Supplementary file 1

Figure S1. Overview of the Super@ intervention (i.e., implementation of the MasterMind project in Badalona site, Spain). [a]MINI: Mini-International Neuropsychiatric Interview, [b]CSQ: Client Satisfaction Questionnaire, [c]SUS: System Usability Scale.

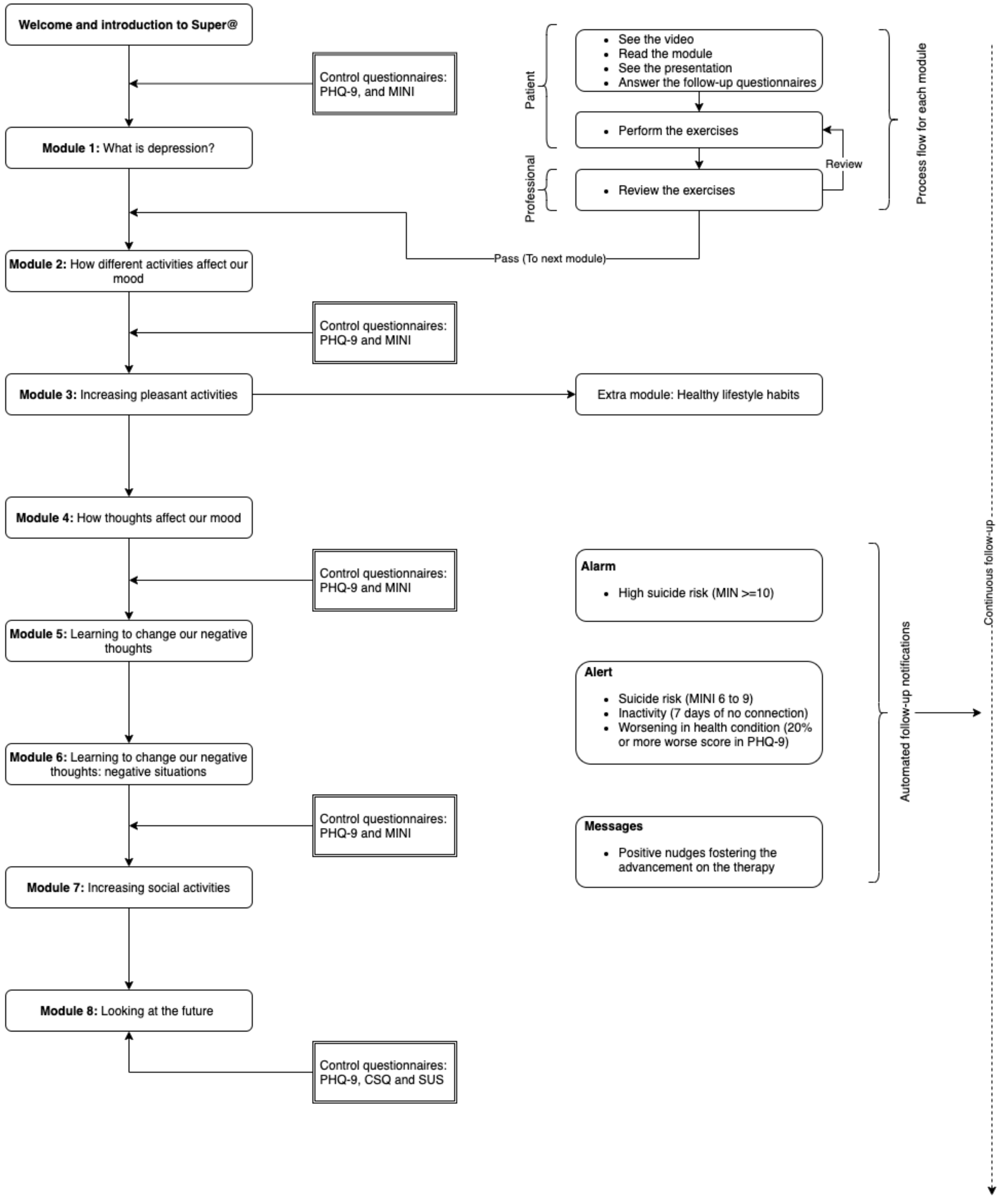

Table S1. Main activities in the project and professional roles involved.

Activity	Description of the activity	Professional role involved
**Recruitment**	Participant identification, fulfilment of eligibility criteria and signature of informed consent form	Health care specialist (mainly GPs in primary care, but also psychiatrists in specialised and intermediate care)
**Inclusion**	Introduction to the intervention and recruitment	Health care specialist
**Coaching**	Introduction to the technology and provision of username and password	Primary Care Nurse
**Follow-up and notification management**	Follow-up on patient performance through the platform and management of automated notifications generated by the technological platform	For all alarms (high suicide risk) and alerts (suicide risk, inactivity, and deterioration of the health condition:<br>- Health care specialist receives the alarm<br>- Project management team receives the alarm (follow-up in case the health care specialist is not responding

Figure S2. Formula for estimating utility of the remission state based on the scores of the Patient Health questionnaire (PHQ-9) questionnaire. **BL**, baseline; **MDS**, minor depressive symptoms (PHQ9 ≥ 5 and ≤ 9); **R**, remission (PHQ9 ≤ 4).

$$Weighted\ utility\ average\ [Remission\ state] = \frac{(Utility\ R)\ x\ (No.patients\ BL\ R) + (Utility\ MDS)\ x\ (No.patients\ BL\ MDS)}{No.patients\ BL\ R + \ No.patients\ BL\ MDS}$$

Figure S3. Patient flow-chart of the MasterMind study in Badalona site (Spain).

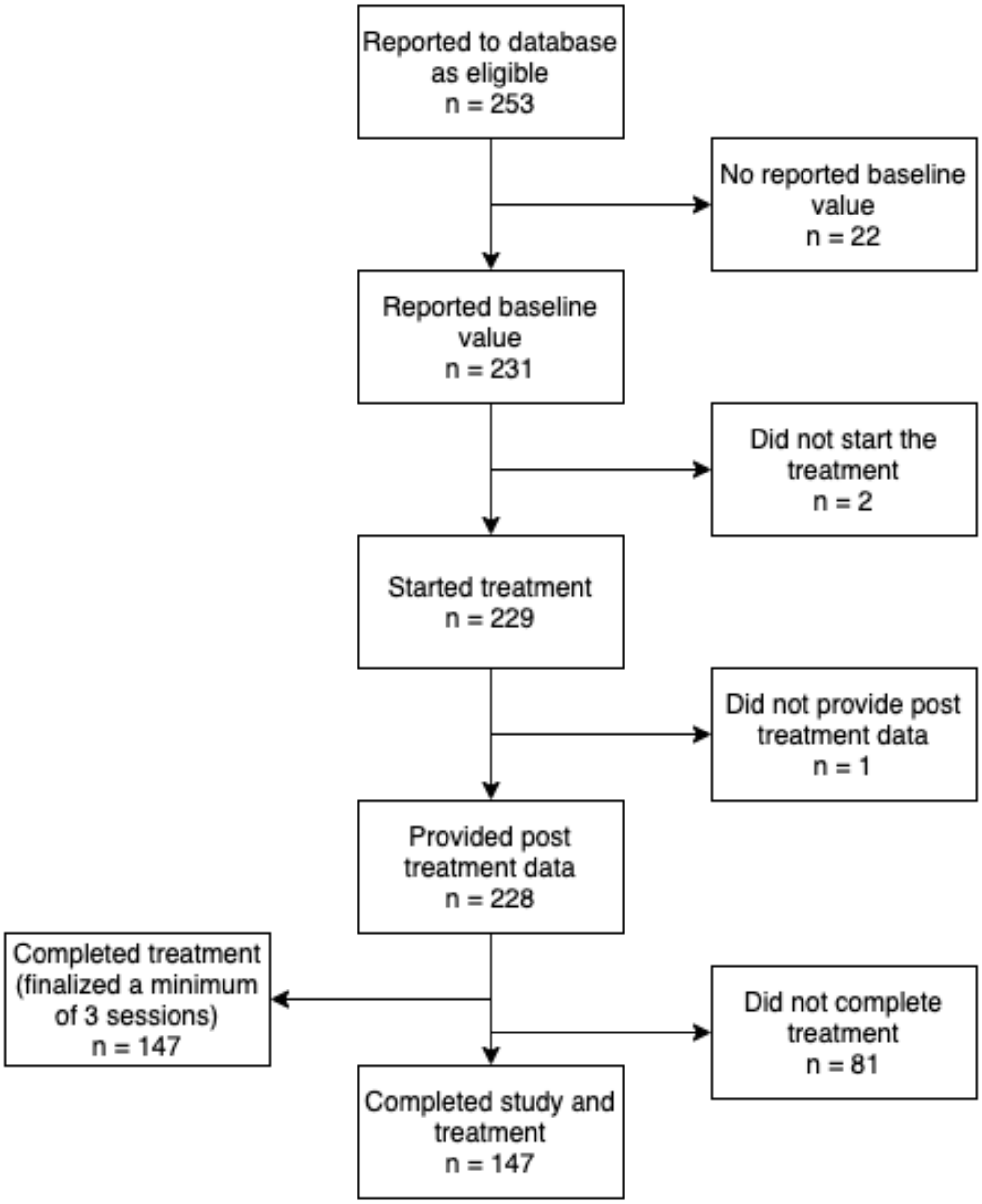

Supplement: Multimedia Appendix 1 [file jmir_v23i5e27410_app1.pdf]
